# Assessment of Exposure to Benzene Among Gasoline Station Workers in Thailand: Risk Assessment Matrix Methods

**DOI:** 10.3390/ijerph22030397

**Published:** 2025-03-08

**Authors:** Sunisa Chaiklieng, Umakorn Tongsantia, Pornnapa Suggaravetsiri, Herman Autrup

**Affiliations:** 1 Department of Occupational Safety and Environmental Health, Faculty of Public Health, Khon Kaen University, Khon Kaen 40002, Thailand; 2 Faculty of Public Health, Khon Kaen University, Khon Kaen 40002, Thailand; 3 Institute of Public Health, Aarhus University, 8000 Aarhus, Denmark

**Keywords:** biomatrix, benzene, occupational safety, risk matrix, tt-muconic acid

## Abstract

This study of risk assessment of gasoline station workers was performed by using the following three models: the occupational safety and health (OSH) risk assessment aligned with ISO 45001, the biomatrix of health risk, and the benzene risk matrix assessment for gasoline station workers. Levels of inhaled air benzene and urine tt-muconic acid (tt-MA) were measured using samples collected from 151 gasoline station workers. Opportunity levels of benzene exposure were obtained by multiplying the frequency of benzene exposure by the levels of tt-MA, the inhaled benzene concentration levels, or the likelihood levels from contributing risk factors at gasoline stations. The final risk scores were calculated by multiplying the opportunity levels by the severity based on the adverse symptoms of benzene toxicity experienced by workers. A checklist regarding risk factors contributing to benzene exposure was used to collect data on occupational safety performance. The potential health risk was at an unacceptable level for 66.23%, 75.50%, and 60.26% of workers according to the OSH risk, the biomatrix of health risk, and the benzene risk matrix model, respectively. There was a significant linear relationship between the risk levels indicated by the three matrix models (r > 0.6, *p* < 0.001). These findings demonstrate that alternative risk assessments can be provided and simply used for preventive action against health hazards from benzene exposure in risk management programs.

## 1. Introduction

Occupational exposure to benzene has effects on the health of gasoline station workers, which have been reported as health problems among gasoline workers. The adverse symptoms, which have been reported as irritation to the skin, eyes, and respiratory system, become more severe through high-level and long-term exposure, with adverse health effects such as headache, dizziness, fatigue, blurred vision, and leukemia, which is the most severe [1,2]. There have been previous studies on the carcinogenic risk associated with long-term exposure to benzene in ambient air at gasoline stations, as well as the emissions from fueling stations in Thailand [3,4,5]. A significantly increased risk was identified among refueling workers positioned in high-risk areas near gasoline dispensers in comparison to cashiers. Moreover, workers at gasoline stations located in urban environments were found to face a higher risk than those employed at stations in rural areas [6,7,8].

The risk assessment mentioned in those studies was estimated and derived from quantitative risk assessment, which did not consider adverse symptoms or their severity levels at the time of workers’ exposure to benzene or their experience of symptoms from past exposure. Accordingly, an occupational health and safety risk management program aligned with international standard ISO 45001 [9] requirements is mostly used in the industrial and manufacturing sectors to certify safety standards [9,10]. The factors found to affect benzene exposure were safety training experience and working as refueling service workers who were close to the fuel dispensers during work [11]. In addition, there have been studies of biomarkers that exhibit abnormalities of hematology and biochemistry, for example, reduction in red blood cell (RBC) counts or increase in white blood cell (WBC) counts, and alterations of serum glutamic pyruvic transaminase (SGPT) or serum glutamic oxaloacetic transaminase (SGOT) among gasoline station workers [12,13]; these studies have reported that exposure to benzene affects the health of gasoline station workers. In addition, other studies of health effects of benzene exposure have also found adverse symptoms: headache, wheezing, dizziness, cracked and dry skin, sore throat, chest pain, blurred vision, cramps, confusion, muscle weakness, depression, anemia, and lung cancer [14,15,16]. Therefore, health surveillance programs, which must be suitable for each workplace according to human resources, budget, and time, are required. The previous studies have been conducted on risk assessments performed by considering the adverse symptom level, the biomarker of exposure (trans, trans-muconic acid: tt-MA, a metabolite of an internal dose of benzene) level compared with the biological exposure index (BEI), which was reliable in occupational exposure screening of workers [3,11,13], and the inhaled benzene concentration level compared with the occupational exposure levels (OELs) set by NIOSH [17]. Therefore, to cover the applied risk assessment based on the requirements of ISO 45001 together with the assessment of the risk of benzene exposure of gasoline station workers, this study aimed to investigate risk assessment in relation to three methods of risk assessment, namely, the occupational safety and health (OSH) risk assessment based on ISO 45001 requirements, the biomatrix of health risk assessment, and the external dose of benzene exposure risk assessment, and use them as a method of health risk assessment on benzene exposure among gasoline station workers. The results of the assessment and parameters affecting benzene exposure will lead to the production of tools for the health surveillance of gasoline station workers.

## 2. Materials and Methods

### 2.1. Sample Size

The sample size calculation gave a final number of 151 workers from 47 gasoline stations. This calculation was performed by following the proportional estimation based on the health risk assessment from a previous report, and the proportion of gasoline station workers who had adverse symptoms from benzene exposure from that previous study [8] (*p* = 0.510, z =1.96, d = 0.05):

*n* = (1.96)^2^ (0.510) (1 − 0.510)/(0.05)^2^
*n* = 150.53, or 151 workers.

The final number of 151 workers met the following inclusion criteria: (1) being aged more than 18 years, (2) having more than 3 months’ work experience at a gasoline station, (3) being able to communicate (read and write) in Thai, and (4) participating voluntarily, and the following exclusion criteria: (1) being pregnant, and (2) smoking or drinking alcohol 24 h before entering into the study. All included workers were monitored for the tt-MA biomarker and inhaled air benzene concentration.

The study was approved by the Khon Kaen University Ethics Committee in Human Research (no. HE612030; date of approval: 12 March 2018). All participants provided informed consent to participate in this study and could leave the study at any time.

### 2.2. Adverse Health Effects (Severity) in the Past 3 Months

The characteristics of exposure to benzene were assessed through an interview questionnaire and divided into five levels, namely, no symptoms, mild, moderate, severe, and very severe, which are described as follows [8]:

Level 1 (No symptoms or non-symptomatic): smell perception;
Level 2 (Mild or low level of symptoms): headache, exhaustion/fatigue, dizziness, red eyes/burning eyes, nasal congestion, runny nose, sore throat/dry throat, suffocation, cough/hoarseness, dry skin, cracked skin, skin rashes/blistering, breathlessness, sleeplessness, and palpitations;
Level 3 (Moderate level of symptoms): muscle weakness/numbness, drowsiness, tight chest, vomiting, blurred vision, cramps, nausea, anorexia, depression, confusion, chest pain, unusual tiredness, tremors, scurvy/bleeding;
Level 4 (Severe or high level of symptoms): anemia, tachycardia, petechia, convulsions, and unconsciousness;
Level 5 (Very severe or very high level of symptoms or diseases): leukemia or other cancer.

### 2.3. Risk Assessment Matrix Methods

#### 2.3.1. Occupational Safety and Health Risk Assessment by ISO 45001 Application

The ISO 45001 method was applied by considering the opportunity level and the severity level of adverse effects with the highest reported symptom levels. The opportunity of benzene exposure level was applied from the following 10 elements, or risk factors, contributing to benzene exposure and collected by using a checklist on occupational safety performance: (1) number of workers, (2) frequency of exposure, (3) workplace measurement, (4) work instruction, (5) training required, (6) standard compliance control, (7) PPE, (8) tool and equipment control, (9) preventive maintenance of tools and equipment, and (10) safety warning signage. All these elements concerned with the opportunity of benzene exposure were considered according to weighted scores, as shown in Table 1. The opportunity of exposure level was calculated as follows.

Opportunity (%) = [Total score (score × weight) × 100] / Total score (full score)

#### 2.3.2. Consideration of the Opportunity of Exposure Level

The opportunity levels, which were based on the occupational health risk assessment with ISO 45001 application scores, were divided into three levels: low level, with a score lower than 50%; medium level, with a score of 50–75%; and high level, with a score higher than 75%. The final OSH risk matrix scores, which were calculated by multiplying the opportunity level by the adverse effects level, were divided into five levels, as shown in Table 2.

#### 2.3.3. The Health Risk Biomatrix Assessment on Benzene Exposure [3]

The reference method from a previous study on gasoline station workers by Chaiklieng et al. [3] considered the opportunity level of benzene exposure via detected tt-MA levels and classified risk levels according to the BEI level [13] (level 1: tt-MA < 10.0% BEI), level 2: 10.0–49.9% BEI, level 3: 50.0–74.9% BEI, level 4: 75.0–100.0% BEI, and level 5: >100% BEI) multiplied by the frequency of exposure, which was extracted from face-to-face interview data and classified into five levels: level 1: once a month, level 2: once a week, level 3: once per work shift (less than 2 h), level 4: continuously for between 2 and 7 h per work shift, and level 5: continuously for 8 h or more per day. The risk scores from the resulting biomatrix (Chaiklieng et al. [3]) consisting of the opportunity level and the severity level were used to classify the risk level of benzene exposure.

#### 2.3.4. The Risk Assessment on Exposure to Benzene

The external dose of exposure risk assessment was based on the reference method from previous studies of Chaiklieng et al. [3]. The opportunity level of exposure according to benzene concentration, which was classified into five levels by comparing it to the OEL-TWA set by NIOSH [17]: level 1 (non-exposure): <10.0% OEL-TWA, level 2 (low): 10.0–49.9% OEL-TWA, level 3 (medium): 50.0–74.9% OEL-TWA, level 4 (high): 75.0–100.0% OEL-TWA, and level 5 (very high): >100% OEL-TWA), was multiplied by the frequency of exposure level (same as the biomatrix level).

The severity of adverse health effects in the past 3 months was assessed by applying the adverse symptom levels (see 2.2) from our previous studies. The risk assessment matrix of the opportunity scores and the severity scores resulted in the matrix levels of benzene exposure shown in Table 3.

### 2.4. Statistical Analysis

STATA version 14 software (StataCorp LLC, College Station, TX, USA) was used for data analysis, and the linear correlation between risk assessment levels according to the Pearson correlation coefficient was significant at *p* < 0.05.

## 3. Results

### 3.1. Characteristics of Gasoline Station Workers

There were 151 gasoline station workers who participated in this study; the average age was 34 ± 9.97 (SD) years, and the min-max was 19–67 years. The mean income (±SD) was 10,634.47 ± 3407 Thai baht per month (min, max = 7000, 25,000). Their average work experience (±SD) was 3.4 ± 4.8 years (min, max = 3 months, 30 years), and their average working hours (± SD) was 8.95 ± 1.12 h per day (min, max = 8, 10 h). The majority of workers were fueling workers (*n* = 117, 77.48%). Those workers worked at gasoline stations located in suburban areas (53.64%) which were situated along Mittraphap Road, the main highway connecting Bangkok, the capital city of Thailand, to other ASEAN countries, in particular the Laos PDR, a neighboring country to the northeast. Other personal characteristics are presented in Table 4.

### 3.2. Opportunity of Benzene Exposure

The biomatrix of exposure and assessment of the external dose of exposure to benzene were used on the basis that the gasoline station workers had a working period of 8 h per day or more. The results showed that all of the workers had a high (level 4: continuous exposure of 4–8 h/day) or very high (level 5: continuous exposure of more than 8 h per day) frequency of exposure. There were 75 fueling workers (64.10%) and 17 cashiers (50.00%) who had a very high frequency of exposure. When considering the working area, it was found that suburban workers (59 workers, 72.84%) had a very high opportunity level, and they were followed by rural workers (25 workers, 59.52%) and urban workers (8 workers, 28.57%), respectively.

### 3.3. Adverse Health Effects (Severity of Symptoms)

Among the 151 workers, there were 90 workers (59.60%) who had adverse symptoms from benzene exposure and 61 workers (40.40%) who reported no adverse health effects. The top five symptoms were headache (40 workers, 74.07%), followed by dizziness (34 workers, 62.96%), exhaustion (34 workers, 62.96%), itchy skin/skin rashes/blistering (32 workers, 59.26%), and nasal congestion (21 workers, 38.89%), respectively. Among the fueling workers, it was found that 66 workers (56.42%) had symptoms of benzene exposure, and the top five symptoms were headache (30 workers, 45.45%), followed by exhaustion (26 workers, 39.39%), itchy skin/skin rashes/blistering (26 workers, 39.39%), dizziness (24 workers, 36.36%), and sore throat/dry throat (16 workers, 24.24%), respectively. Among the cashiers who had symptoms of benzene exposure (24 workers, 70.59%), the top five symptoms were itchy skin/skin rashes/blistering (12 workers, 50.0%), followed by headache and dizziness (10 workers, 41.67%), exhaustion/fatigue (8 workers, 33.33%), and nasal congestion (6 workers, 25.0%).

The most frequent symptoms in the moderate-to-severe range were tight chest, numbness, and scurvy/bleeding. The severity of adverse effects experienced in the last 3 months was classified into four levels, namely, no symptoms (*n* = 61; 40.4%) and mild (*n* = 54; 33.77%), moderate (*n* = 33; 21.85%), and severe (*n* = 3; 1.99%) symptoms, respectively.

In the case of the workers who had mild symptoms, the proportion of cashiers with mild symptoms was higher than the proportion of fueling workers with the same level of symptoms. The number of cashiers who had mild symptoms was 16 workers (47.06%), and the number with symptoms at the moderate level was 8 workers (23.53%). The number of fueling workers with symptoms at the mild level was 38 workers (32.48%), the number with symptoms at the moderate level was 25 workers (21.38%), and the number with symptoms at the severe level was 3 workers (2.56%).

### 3.4. Risk Assessment Matrix Results

#### 3.4.1. Occupational Safety and Health Risk Assessment Performed by Applying ISO 45001

The opportunity levels of benzene exposure among gasoline station workers, according to the 10 elements, were calculated as weighted scores. The results showed that most of the workers had a low opportunity of exposure (116 workers, 76.82%) or a moderate opportunity of exposure (35 workers, 23.18%). The mean average opportunity score was 52.74, the median (min, max) was 53.09 (43.21, 70.37), and the 95th percentile was 67.90.

Regarding job functions, the highest proportions of fueling workers (27 workers, 23.08%) and cashiers (8 workers, 23.53%) had an opportunity of exposure which was at a moderate level. When considering the locations of gasoline stations, namely, urban, suburban, and rural areas, it was found that the highest proportions of workers had an opportunity of exposure which was at the moderate level, with 1 worker (3.57%), 19 workers (23.46%), and 15 workers (35.71%) at this level, respectively.

There were four levels of risk assessment reported, which were trivial (51 workers, 33.77%), tolerable (51 workers, 33.77%), moderate (37 workers, 24.50%), and substantial (12 workers, 7.95%). The highest proportion of fueling workers had a trivial risk (43 workers, 36.75%), while the highest proportion of cashiers had a tolerable risk (14 workers, 41.18%). Those working in urban and suburban areas had a tolerable risk level, with 11 workers (39.24%) and 33 workers (40.74%) at this level, respectively. Regarding stations in rural areas, it was found that the highest proportion of workers had a moderate level of risk (12 workers, 28.57%). There was a significant correlation between location and risk level (*p* = 0.014).

#### 3.4.2. Biomatrix of Health Risk Assessment

The risk assessment of benzene exposure among gasoline station workers was performed by considering the exposure opportunity based on the frequency of exposure and the level of tt-MA in urine at the end of shiftwork multiplied by the severity of symptoms.

Regarding the detection level of the tt-MA biomarker of benzene exposure, the average concentration of tt-MA found among gasoline workers was 473.28 ± 792.48 ug/g creatinine (Cr), and the maximum was 5986.44 ug/g Cr. The detected tt-MA was classified into five levels of concentration range, as shown in Table 5.

The biomatrix of health risk results showed that most of the workers were at a low risk (88 workers, 58.28%), followed by those at an acceptable risk level (37 workers, 24.50%) and those at a moderate risk level (26 workers, 17.22%). With regard to the type of work, most fueling and cashier workers had a low risk, with 67 workers (57.26%) and 21 workers (61.76%) at this level, respectively, followed by those with a moderate risk, with 21 workers (17.95%) and 5 workers (14.71%) at this level, respectively. When considering the gasoline station areas, it was found that most workers in all areas were at a low risk level, with those of the urban, suburban, and rural areas at that level comprising 16 workers (57.14%), 44 workers (54.32%), and 28 workers (66.67%), respectively, while those at a moderate risk level comprised 6 workers (21.43%), 13 workers (16.05%), and 7 workers (16.67%), respectively.

#### 3.4.3. Benzene Risk Matrix Assessment

The average inhaled benzene concentration of workers exposed to benzene at gasoline stations located across three zones (urban, suburban, and rural) in Khon Kaen province, Thailand, was 10.83 ± 16.59 ppb. The maximum concentration was 136.9 ppb, and the detected concentrations were classified into three levels, as shown in Table 6.

According to the benzene risk matrix assessment, most of the gasoline station workers had an unacceptable level of risk (91 workers, 60.26%), comprising 67 refueling workers (57.26%), and 24 cashiers (70.59%), and 39.74% of workers had acceptable risk. The risk assessment results showed that the highest proportion of workers with this level of risk were urban workers (22 workers, 78.59%), followed by rural workers (24 workers, 59.52%) and suburban workers (44 workers, 54.32%), respectively. When considering the highest severity of benzene toxicity, or cancer (level 5), all workers were at an unacceptable level of risk.

#### 3.4.4. The Linear Correlation Between Risk Levels from All Risk Assessment Methods

**Figure 1:**
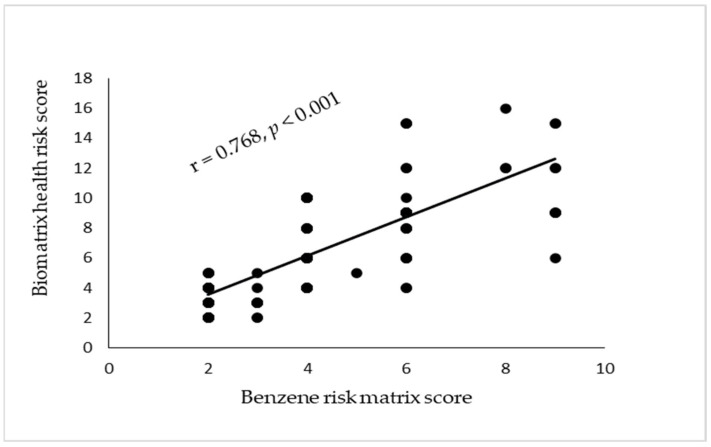
The correlation between the biomatrix of health risk score and benzene risk matrix score. The significance was identified at *p* < 0.001, and the Pearson’s correlation coefficient (r) was 0.768.

The risk levels of the biomatrix of health risk assessment are correlated with the risk levels of the benzene risk matrix assessment and assessment with ISO 45001 application, with statistical significance at *p* < 0.001 (Fisher’s exact test). The risk levels of the benzene risk matrix assessment and assessment with ISO 45001 application are correlated with statistical significance at *p* < 0.001. Risk assessment of benzene exposure with ISO 45001 application had risk level distribution across all levels (acceptable, low, moderate, and high). Most of the workers were at low or moderate risk levels (33.77% and 24.5%). This correlation is presented in Figure 1.

**Figure 2:**
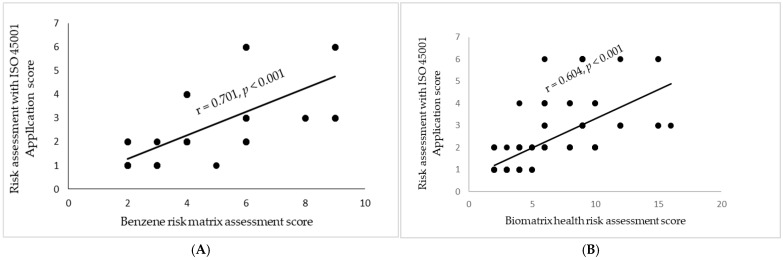
The correlations between risk assessment with ISO 45001 application score and benzene risk matrix score (**A**) and the biomatrix of health risk score (**B**). Significance was identified at *p* < 0.001, and the Pearson’s correlation coefficient (r) values were 0.701 and 0.604 for external dose (benzene) risk score (**A**) and biomatrix of health risk score (**B**), respectively.

Regarding the biomatrix of health risk assessment and the benzene risk matrix, it was found that most of the workers had a low level of risk according to the two models (58.28% and 60.26%). The comparison of the three methods showed that each risk assessment resulted in workers having a similar risk level, which was confirmed by the statistically significant correlation of risk scores between the biomatrix of health risk model and the risk matrix of benzene exposure (r = 0.768, *p* < 0.001), between the risk matrix of benzene exposure and the risk assessment with ISO 45001 application (r = 0.701, *p* < 0.001), and between the biomatrix of health risk model and the risk assessment with ISO 45001 application (r = 0.604, *p* < 0.001). These correlations are presented in Figure 2A,B.

## 4. Discussion

The study showed that most gasoline station workers who were affected by benzene exposure had the highest level of adverse effects on health (severe symptoms) and had a moderate opportunity of potential exposure. According to the results on the symptoms of workers, there was a proportion of workers who had symptoms of benzene toxicity (68.4%) which was higher than the proportion found in a previous study on gasoline station workers, which was about 60% of workers [8,18]. There were many symptoms of benzene exposure, which can occur in the respiratory system, digestive system, nervous system, and skin; however, the symptoms found in this study were mostly neurological symptoms, i.e., headache, dizziness, and exhaustion. This was consistent with previous studies related to symptoms caused by benzene exposure, which found that the affected nervous system resulted in similar symptoms to those found in this study.

Gasoline station workers and other workers who are exposed to benzene in ambient air also often experience first-order neurological symptoms [19]. From the assessment of severity, it was found that symptoms that occurred in most gasoline station workers were at the mild level of severity, while there was also a number of workers with symptoms at the moderate level of severity. Those with a moderate-to-high level of severity were found to be cashier workers, which was consistent with the severity and risk assessment of a previous study of gasoline station workers. The explanation was that a cashier was more likely to be permanently sitting in an area close to fuel than a fueling worker, without any change in position. Therefore, some cashiers were exposed to benzene, affecting the severity level of adverse health effects [11].

Semiquantitative risk assessments, namely, the biomatrix risk assessment and the inhaled benzene risk matrix have been used for risk assessment of exposure to benzene [3]. These assessments produced similar data sets, where the only difference was the concentration levels of chemicals in the working ambient air in which employees were exposed to benzene. In this study, it was found that gasoline station workers were exposed to higher concentrations (0.1–136.9 ppb) than before, when a range of 0.03–65.7 ppb [11,18] was observed. The benzene concentration found in urban stations indicated that fueling workers were more exposed to benzene in such areas, supporting the previous studies that compared the concentration in an urban area to the concentrations in suburban and rural areas [5,8]. In addition, the concentration that the fueling workers were exposed to was higher than the concentration that cashiers were exposed to, which is similar to the finding of a previous report comparing office-based policemen with traffic policemen [20].

The three risk matrix assessment methods, which were the OSH risk assessment with the application of the ISO 45001 matrix, the health risk biomatrix, and specific benzene risk matrix models, demonstrated an unacceptable risk level, where the fueling workers had a higher level of risk than the cashiers. Therefore, the risk assessment results of this study showed an unacceptable level of risk which was four times higher than that of a previous report [3].

Between 2020 and 2023, the number of cars using automotive gasoline, including benzene chemical mixture, increased by around 1.37% [21] due to the economic situation stimulating an increase in travel. As a result, the number of gasoline service stations has increased, and the demand for services from drivers has increased; this is in accordance with studies that showed that benzene concentrations in the working atmosphere had increased in some areas of Thailand. Benzene concentrations in those areas were found to be higher than the recommended standard [13], such as in Rayong province, where the Map Ta Phut Industrial Estate is located [22].

Risk assessment based on the detected levels of the tt-MA biomarker was divided into five levels to be used as a basis for categorizing exposure based on criteria from a previous study on gas station employees [3], which found that 36.70% of the gasoline workers were at an unacceptable risk level. The proportion (75.50%) of workers at an unacceptable level of health risk when considering the tt-MA biomarker level in the risk assessment matrix on workers’ exposure to benzene was two times higher. A study of tt-MA levels among gasoline workers exposed to benzene in other areas of Thailand also found that exposure levels were higher [4,8] because the frequency of exposure in the past was based on different criteria to the present. It was found that gasoline workers in Thailand had a high level of frequency of exposure because the working period is legally limited to a maximum of 48 h per week and 8 h per day, and overtime (OT) work is permitted for no more than 3 h per day, which is equal to 11 working hours per day [23].

From assessing the health risks of exposure to benzene by using the external dose of benzene exposure and the internal tt-MA biomarker dose of exposure, it was found that the health risks of workers at gasoline stations were therefore higher when compared to those of the past study on exposure and when the safety measures and performance data of each gasoline station were considered. In accordance with the international standard for occupational safety and working environment management guideline (ISO 45001), safety performance information and frequency of exposure were used together with the experience of adverse symptoms elicited from an interview to calculate the level of potential exposure, and it was found that occupational exposure to benzene had the same effects as those found by using the two health risk assessment models above.

It was found that the majority of employees were at an unacceptable level of risk (66.23%). This risk assessment included 7.95% of employees at high risk, indicating that employees working at fuel stations lacked control in terms of occupational health and safety and were at high risk from working with benzene. From previous studies related to the introduction of safety measures or the ISO 45001 system into management so that employees would work safely, it was found that the top management teams of Indonesian industries and the explosives manufacturing industry believed that ISO 45001 had a significant effect on employee performance [24,25].

From the results of the three methods of risk assessment, it can be seen that the risk levels were similar even though the opportunity levels considered were from different data sources. However, risk assessment in accordance with the ISO 45001 format is a risk assessment that has the lowest required budget for data preparation and does not require consent to collect employee urine for analysis of biomarker levels in the body. It is also a risk assessment that takes the least amount of time in terms of analysis of monitoring results. Therefore, it is recommended that executives or personnel involved in safety operations at fuel service stations adopt a risk assessment model using ISO 45001 guidelines to assess the health risks of fuel service station staff. In addition, it can be used as a guideline for preventing dangers and occupational diseases caused by exposure to benzene in the workplace. This recommendation of following ISO 45001 guidance to reduce risk by installing safety equipment, namely, a vapor recovery system (VRS), is in line with a study of benzene exposure in gasoline station workers in Thailand and Mexico in gasoline refueling stations with a VRS. The study found that the workers of gasoline stations where investment had been made to install a VRS had risk reductions of 47–60% compared to those working in stations without installation of VRS [26].

This investigation was conducted in areas located along the Mittraphap Road, the main highway of Khon Kaen province directly connected to Bangkok, the capital city of Thailand, and to ASEAN countries to the north and northeast of Thailand. Therefore, the promotion in investment for VRS installation at fuel dispensers in high-risk gasoline stations along this main route could impact not only the economic growth figure for customer services [7] but may also result in a health risk reduction from lower VOC pollution exposure among customers.

The strength of this study’s method is that, firstly, tt-MA biological monitoring was done by following the standard method, and analysis of the biomarker of exposure was performed in a certified laboratory, and secondly, an appropriate risk assessment matrix method based on the specific exposure profiles of the gasoline workers was provided by considering efficiency and budget in relation to the models. The limitation of this study is that the health screening was performed by conducting an interview on adverse symptoms experienced in the last 3 months, which could result in possible recall bias due to the collection of retrospective data. Therefore, confirmation through medical screening for severity is suggested for the health surveillance program.

## 5. Conclusions

The study showed that most gasoline station workers who were affected by benzene exposure had the highest level of adverse effects on health (severe) and had a moderate opportunity of potential exposure. The three risk matrix assessment methods, which were the OSH risk assessment with the application of the ISO 45001 matrix, the health risk biomatrix, and the specific benzene risk matrix models, demonstrated an unacceptable risk level, where the fueling workers had a higher level of risk than the cashiers. All risk assessment methods indicated a significant linear relationship between risk levels (r > 0.6, *p* < 0.001). The workers in urban areas had higher exposure to benzene than those in the suburban and rural areas. Therefore, the enterprise owners of gasoline stations should be concerned about effective risk management programs and choose an appropriate risk matrix method based on the specific exposure profiles of their workers by considering efficiency and budget in relation to a workers’ health surveillance program. In addition, engineering control of VRS installation should be considered to reduce risk by following the ISO 45001 guidance.

## Figures and Tables

**Table 1:** Opportunity rating criteria of OSH risk aligned with ISO 45001.

No.	Opportunity Rating Criteria	Weighted Score
1	The number of workers exposed (>10 persons = 3, 6–10 persons = 2, 1–5 persons = 1)	3
2	Frequency of exposure (>30 h/week = 3; 10–30 h/week = 2; <10 hrs./week = 1)	3
3	Workplace measurement (no measurement = 3; measured but exceeding legal standards = 2; measured and compliant with legal standards = 1)	3
4	Standard work instruction/procedures (no procedure documents = 3; there are work instruction documents, but they are not suitable for the level of risk = 2; there are work instruction documents/procedure documents and they are suitable for the level of risk = 1)	3
5	Effective training instruction/procedures (uncontrolled training = 3; there is controlled training, but it is discontinuous = 2; there is training with continuous control = 1)	3
6	Control of compliance with standard procedures (uncontrolled = 3; controlled but discontinuous = 2; continuous control = 1)	2
7	Appropriate PPE/usage control (no = 3; yes, but inappropriate = 2; proper and continuous control = 1)	2
8	Proper usage of tools, machinery, equipment, and safety equipment (no = 3; yes, but inappropriate = 2; usage designed with proper safety protection = 1)	3
9	Maintenance of tools and equipment (no maintenance = 3; maintenance, but no record = 2; maintenance and continuous recording = 1)	3
10	Proper warning and safety signage (no warnings = 3; warnings, but not suitable for risk characteristics = 2; proper warnings suitable for risk characteristics = 1)	2
	Total (Full Score)	81

Applied from Chaiklieng et al. [18]. Note: criteria for opportunity rating with relevant scoring.

**Table 2:** The application of OSH risk assessment according to ISO 45001.

Opportunity Level	Severity		
	1: Mild	2: Moderate	3: Severe
3: High	Moderate (3)	Substantial (6)	Intolerable (9)
2: Moderate	Tolerable (2)	Moderate (4)	Substantial (6)
1: Low	Trivial (1)	Tolerable (2)	Moderate (3)

Applied from Chaiklieng et al. [18].

**Table 3:** Exposure to benzene and risk assessment.

Adverse Health Effect Level	Likelihood of Exposure Level (Five Levels of Inhaled Benzene × Five Levels of Exposure Hours)						Risk Assessment	
	1	2	3	4	5	Score	Risk	Level
5: Very severe	5	10	15	20	25	21–25	Very high	5
4: Severe	4	8	12	16	20	17–20	High	4
3: Moderate	3	6	9	12	15	9–16	Medium	3
2: Low	2	4	6	8	10	4–8	Low	2
1: No symptoms	1	2	3	4	5	1–3	Acceptable	1

Applied from Chaiklieng et al. [3].

**Table 4:** Characteristics of gasoline station workers (*n* = 151).

Characteristics	*n* (%)
Gender	
Male	56 (37.09)
Female	95 (62.91)
Marriage status	
Single	76 (50.33)
Married	64 (42.38)
Education level	
Primary	27 (17.88)
Secondary	60 (39.74)
High school and higher	35 (23.18)
Working position	
Fueling worker	117 (77.48)
Cashier	34 (22.52)
Working location	
Urban	28 (18,54)
Suburban	81 (53.64)
Rural	42 (27.81)

**Table 5:** Levels of the tt-MA biomarker of benzene exposure (*n* = 151).

tt-MA Level (ug/g Cr.)	*n* (%)
≤50	27 (17.88)
>50–100	15 (9.93)
>100–250	39 (25.83)
>250–500	29 (19.21)
>500	41 (27.15)
Median (min, max) = 226.5 (5.32, 5986.44)	

**Table 6:** Inhaled air benzene concentration of gasoline station workers (*n* = 151).

Benzene Concentration (ppb)	*n* (%)
≤25	129 (85.43)
>25–50	17 (11.26)
>50	5 (3.31)
Median (min–max): 4.6 (0.1–136.9)	

## Data Availability

Data are unavailable due to privacy restrictions. Data may be available upon request to the corresponding author depending on the case.
